# Diagnostic Performance of Red Cell Distribution Width in Adult Iraqi Patients with Ankylosing Spondylitis

**DOI:** 10.1155/2018/2904694

**Published:** 2018-08-05

**Authors:** Faiq I. Gorial, Ali M. Hassan

**Affiliations:** ^1^Rheumatology Unit, Department of Medicine, Collage of Medicine, University of Bagdad, Iraq; ^2^Baghdad Teaching Hospital, Rheumatology Unit, Baghdad, Iraq

## Abstract

**Background:**

Ankylosing spondylitis (AS) is a chronic, progressive inflammatory rheumatic disease that leads to structural damage, functional impairment, and decrease in the quality of life. Red cell distribution width (RDW) is a part of the complete blood count (CBC) and estimates erythrocyte variability.

**Objective:**

To analyse RDW in patients with AS and to evaluate the relationships with acute phase reactants (APRs) and disease activity index.

**Patients and Methods:**

A total of 100 patients with AS (78 males and 22 females) were diagnosed according to the modified New York classification criteria for AS and 146 (99 males: 47 females) healthy individuals matched in age and sex as controls enrolled in the study. Demographic data, disease activity scores using Bath Ankylosing Spondylitis Disease Activity Index (BASDAI), medical history, C-reactive protein (CRP), erythrocytes sedimentation rate (ESR), and complete blood count (CBC) were measured.

**Results:**

The mean age for patients and controls was 38.0 ± 9.0 and 35.8 ± 9.0 years, respectively (p=0.057). RDW was significantly higher in patients with AS compared with controls (14.133 ± 1.613 versus 12.299 ± 1.031, p < 0.001). There was a direct correlation of RDW with both ESR and CRP (P < 0.001); RDW had r=0.38 for C-reactive protein (CRP) and r=0.413 for ESR. Also BASDAI was directly correlated with RDW (r=0.326 p<0.001). RDW was a valid measure to differentiate between patients with AS and controls (AUC=0,84, p<0.001) and at optimum cut-off value>13% has highest accuracy (78.9%) with very good sensitivity test (81%) and NPV (85.6%) as well as good specificity (77.4%) and PPV (71.1%).

**Conclusion:**

RDW was higher in AS patients compared with controls and was directly correlated with ESR, CRP, and BASDAI. RDW was a valid simple measure with good accuracy to differentiate between patients with AS and controls.

## 1. Introduction

Ankylosing spondylitis (AS) is a chronic, progressive inflammatory rheumatic disease that affects the axial skeleton causing characteristic back pain, structural and functional impairment, and decrease in the quality of life [1].

In Iraq, the estimated prevalence of AS is 0.13%, where 84% are HLA-B27 positive, while 2.1% of healthy populations are HLAB27 positive [2, 3]. The collective impact of AS has a substantial influence on patients' quality of life. Over 75% of patients are able to remain in employment and enjoy a good quality of life [4].

The RDW is a parameter of complete blood count (CBC) [5, 6]. RDW is a numerical measure that describes red blood cell volume heterogeneity and serves as a component of complete blood count in the differential diagnosis of anaemia [7].

In fact, RDW has been shown to be strongly associated with CRP and ESR in a large cohort of unselected outpatients [8]. In addition, recent studies have shown that RDW was associated with the severity of rheumatoid arthritis (RA) [9], inflammatory bowel diseases (IBDs) [10], and Behcet's disease (BD) [11]. Two new studies had also determined that RDW was increased in patients with systemic lupus erythematosus (SLE) and related with SLE disease activity index (SLEDAI), ESR, and CRP [12].

This study aimed to evaluate serum RDW in patients with AS and to assess its relationships with APRs and AS disease activity index.

## 2. Patients and Methods

### 2.1. Study Design

This observational prospective case-control study was conducted at the Rheumatology Unit of Baghdad Teaching Hospital in Medical City and Basra Center for Biological Therapy in Al-Basra General Hospital from August 2016 to March 2017.

### 2.2. Sample Selection

A total of 100 consecutive patients diagnosed as having AS according to the modified New York criteria [13] were included in the study and were compared with another 146 healthy controls matched in age and sex. The controls were healthy asymptomatic individuals without any previous disease and were not taking any medication and collected from the relatives of other patients with other diagnosis who attended the hospital and from healthy medical staff.

Informed consent was obtained from each participant included in this study according to the declaration of Helsinki. Ethical approval was obtained from the Ethics Committee in Medical Department, College of Medicine, Baghdad University.

Patients were excluded from the study if they had one of the following: other autoimmune diseases such as Sjogren Syndrome (SS), SLE, RA, IBDs, and psoriasis; acute or chronic infection; malignant diseases; end stage renal disease; liver disease such as hepatitis and liver cirrhosis; haematological disorders or receiving blood transfusion during the past 4 months; acute myocardial infarction and cerebrovascular disease; and pregnancy or time of 6 months postpartum.

### 2.3. Data Collection and Entry

Data entry of patients and controls was done using paper clinical research (CRF) form through interview and questionnaires. Patients age, sex, disease duration, and smoking status, body mass index (BMI) according the equation BMI=weight / height ^2^, disease activity and functional class, and medications were recorded. All controls were asked about age, sex, smoking status, height, weight, and BMI.

### 2.4. Methods and Data Monitoring

Blood samples were taken in both groups for measuring complete blood count (CBC), erythrocyte sedimentation rate (ESR), C-reactive protein (CRP), and red cell distribution width (RDW). Disease activity was measured using Bath Ankylosing Spondylitis Disease Activity Index (BASDAI) [14], and functional class was evaluated using Bath Ankylosing Spondylitis Functional Index (BASFI) [15]

### 2.5. Sample Size Calculation

A target sample size of at least 86 patients and 86 controls was calculated to provide approximately 90% statistical power with medium effect size of 50% and *α* error probability of 0.05 as a significant level.

### 2.6. Statistical Analysis

Anderson darling test was done to assess if continuous variables follow the normal distribution; if they followed normal distribution then mean and standard deviation were used and if not then we used median (interquartile range). Discrete variables were presented using numbers and percentages used to present the data. Chi square test was used to analyse the discrete variable or Fisher exact test used to analyse the distribution between 2 groups (used instead of chi square for 2x2 table, if total sample <20 and if 2 or more are with expected frequency less than 5). Two-sample t test was used to analyse the differences in means between two groups—if both follow normal distribution with no significant outlier—and Mann–Whitney test was used if they did not follow the normal distribution.

Linear regression analysis was performed to assess the relationship between different variables; if one or both of them follow normal distribution, person regression will be used but if both did not follow normal distribution spearman correlation will be used. r (correlation coefficient or standardized beta) is a representative of magnitude and direction of the relationship: r<0.25, weak correlation; 0.25–0.5, mild correlation; 0.5–0.75, moderate correlation; >0.75, strong correlation. Negative sign indicates inverse relationship, but positive sign represents direct relationship.

Receiver operating characteristics curve was used to assess the validity of different parameters in separating cases from controls. Area under the curve, i.e., AUC, and its p value described this validity (AUC ≥ 0.9 means excellent test, 0.8–0.89 means good test, and 0.7–0.79 means fair test, otherwise unacceptable). Trapezoidal method was used for calculating the curve.

In ROC curve the true positive rate (sensitivity) is plotted in function of the false positive rate (100-specificity) for different cut-off points. Each point on the ROC curve represents a sensitivity/specificity pair corresponding to a particular decision threshold. A test with perfect discrimination (no overlap in the two distributions) has a ROC curve that passes through the upper left corner (100% sensitivity, 100% specificity).

Statistical software types used for statistical analysis were SPSS 20.0.0, MedCalc 14.8.1, and GraphPad Prism 7.0. P value less than 0.05 was considered statistically significant.

## 3. Results

The mean age (± SD) of patients was 38.0 ± 9.0 years and that of controls was 35.8 ± 8.3 years. Male patients were 78 (78.0%) and male controls were 99 (67.8%). The mean BMI of patients was 28.3 ± 5.5 and of controls was 28.6 ± 3.8 kg/m ^2^. There were no significant differences between patients and controls in age, sex, and BMI. Other baseline characteristics were shown in Table 1.

**Figure 1:**
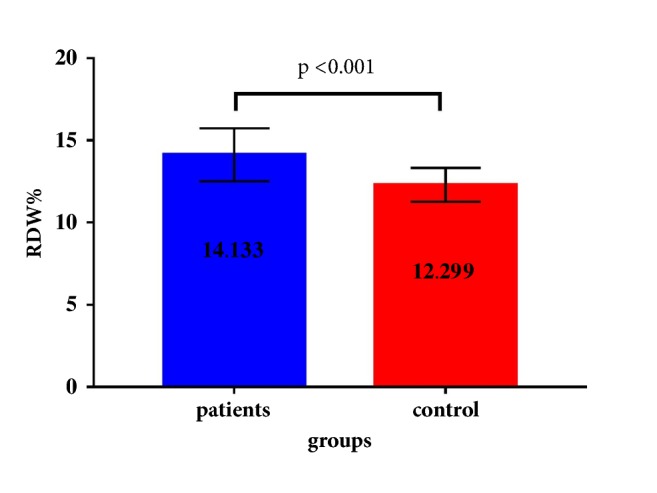
Error bars of red cell distribution width (RDW) in ankylosing spondylitis patients and controls.

The RDW was significantly higher in patients (14.133 ± 1.613%) with AS compared to controls (12.299 ± 1.031%) as in Figure 1. In univariate analysis RDW was significantly and inversely correlated with age and hemoglobin, while it was directly and significantly correlated with BASDI, ESR, CRP, and platelet. In multivariate analysis using stepwise regression method only age (inverse), CRP (direct), and hemoglobin (inverse) were significantly and independently correlated with RDW as illustrated in Table 2.

**Figure 2:**
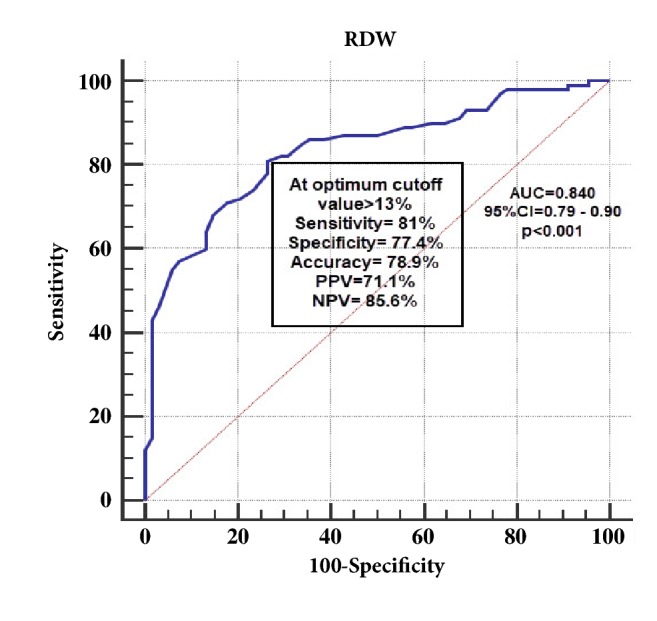
ROC curve showing RDW as a test to differentiate AS patients from controls. AS: ankylosing spondylitis; ROC: receiver operator characteristics; RDW: red cell distribution width; MPV: mean platelets volume; AUC: area under curve; CI: confidence interval; PPV: positive predictive value; NPV: negative predictive value.

The RDW had good ability to differentiate between patients and controls (AUC =0.84, 95%CI= 0.79-0.90, p<0.001). RDW at optimal cut-off point >13.0% had more sensitivity than specificity when used as single test for diagnosis, and its NPV is higher than PPV indicating it can be used as exclusion test when used in conjugation with prior test, as in Figure 2.

## 4. Discussion

This study measured the level of RDW in AS patients in comparison with control group and evaluated different parameters to avoid the cofactors that may influence the results. It showed that RDW was significantly higher in AS patients than in controls. RDW was statistically a good exclusion test when used with appropriate conjugate tests as its NPV (85.60%) was higher than PPV (71.10%) with more sensitivity (> 81.00%) than specificity (77.40%) at the optimal cut-off point > 13.0. Also, RDW was a good test to differentiate AS patients from controls (AUC= 0.84). There was a direct correlation of RDW with CRP (P < 0.001 for RDW) and with BASDI (p<0.001).

The interaction between autoantibodies, genetic factors, and environment is the main trigger of chronic inflammatory process in autoimmune diseases including AS [16]. MRI has confirmed the evidence of correlation of spinal inflammation with AS [17]. In addition, studies have reported that inflammation was associated with tumor necrosis factor (TNF) and interleukin-6 (IL-6) and inflammatory response possibly was participator in the pathogenesis of AS [18, 19]. RDW is a novel inflammatory indicator independent of sex, age, and haematological variation [20].

To the best of our knowledge, there are few studies testing RDW as a diagnostic or prognostic marker for AS. Similar findings were reported by Peng et al. [21] who concluded that RDW levels were significantly different in AS patients compared with healthy controls (AUC= 0.853; sensitivity of 72.7%, specificity of 81.4%) and were positively associated with ESR and CRP levels and BASADI score. They suggested that RDW is a more reliable marker for AS activity than CRP and ESR because it is not affected by acute infection.

The long-term chronic inflammation possibly explains potential correlation between RDW and CRP and ESR in patients with AS. Patients with AS involve chronic inflammation referring to IL-6 and TNF from tendon and attachment points of ligament, which impacts nature of RBC, production of glycoprotein, iron metabolism, the lifespan of RBC, and sensitivity of erythropoietin contributing to increase of RDW [8, 22, 23].

Sezgin et al. [24] demonstrated findings in agreement with the current study. They reported that RDW was significantly higher in AS patients (both active and inactive disease) and it was more in active ones than healthy control individuals with cut-off equal to 14.8%, sensitivity 43.6%, specificity 97.7%, and AUC= 0.76 and was positively correlated with CRP, ESR, and BASDAI. They suggested a possible mechanism of the adverse effects of chronic inflammation on erythropoiesis and thus anisocytosis occurrence via direct erythroid precursor's suppression reduces the production of erythropoietin by kidneys, reduces the iron bioavailability, and induces apoptosis and increases the resistance to erythropoietin of the erythroid precursor cell line.

The difference of the current study in sensitivity/specificity comparison from the previous two studies may be related to the differences in the study design, sample size, and geographical factors.

Limitations of the present study include the time limit (short duration of study) and the financial barrier to do the tests for every AS patient even when clinically not required plus the healthy control individuals tests. Many AS patients who are residents of cities that face war circumstances and even those who reside in quite cities but distant from the study centres are not involved in the study and all these affected not only sample size but also the geographical distribution and coverage. However, this is the first study in Iraq that evaluated RDW as a parameter in the diagnosis of AS and correlation with disease activity. It is a cheap and easy test to be performed as it is a common component of the complete blood count (CBC) test that is nowadays widely distributed in nearly every healthcare facility and by an automated machine.

In conclusion the RDW was significantly higher in AS patients compared with controls and was statistically a good valid and cheap test to differentiate between AS patients and controls. In addition, there was a direct correlation between both RDW with ESR and CRP and BASDAI. Larger sample size and longer duration of study are needed to further validate the findings of this study.

## Figures and Tables

**Table 1:** Demographic and clinical characteristics of patients and controls.

**Variables**	Controls	Patients	P value
**Number**	146	100	-
**Age (mean **±** SD), years**	35.8 ± 8.3	38.0 ± 9.0	0.057
**Sex**			0.081
** Female**	47 (32.2%)	22 (22.0%)	
** Male**	99 (67.8%)	78 (78.0%)	
**BMI**	28.6 ± 3.8	28.3 ± 5.5	0.596
**Smoking**	45 (30.8%)	41 (41.0%)	0.100
**Disease duration**	-	9.0 (5.0 – 13.8)	-
**BASDAI**	-	4.2 ± 1.6	-
**BASFI **	-	4.1 ± 1.6	-
**Biologics **	-	89 (89%)	-
**DMARDs**	-	17 (17%)	-
**NSAIDs**	-	68 (68%)	-
**Steroids **	-	5 (5%)	-
**ESR median (IQR), mm/hr **	10(5.25-15.75)	34 (18 – 50.8)	<0.0001
**CRP median (IQR), mg/dl**	2(1-3)	9 (5 – 15.2)	<0.0001

SD: standard deviation, BMI: body mass index, BASDAI: Bath Ankylosing Spondylitis Disease Activity Index, BASFI: Bath Ankylosing Spondylitis Functional Index, DMARDs: disease modifying antirheumatic drugs, NSAIDs: nonsteroidal anti-inflammatory drugs, ESR: erythrocytes sedimentation rates, CRP: C-reactive protein, and IOQ: interquartile range.

**Table 2:** Multiple linear regression analysis to show the effects of demographic and clinical characteristic on RDW.

**Variables**	Univariate		Multivariate	
	r	P value	r	P value
**Age**	-0.216	0.016 [Sig.]	-0.307	0.002
**BMI**	-0.051	0.309		
**Disease Duration**	-0.165	0.051		
**BASDI**	0.326	<0.001 [Sig.]		
**BASFI**	0.010	0.460		
**ESR**	0.413	<0.001 [Sig.]		
**CRP**	0.360	<0.001 [Sig.]	0.251	0.013
**HB**	-0.486	<0.001 [Sig.]	-0.421	<0.001
**WBC**	-0.013	0.449		
**Neutrophil **	0.002	0.493		
**Lymph**	-0.071	0.243		
**N/L ratio**	-0.013	0.448		
**PLT**	0.227	0.012 [Sig.]		
**PLT/L ratio**	0.023	0.411		
**Monocyte**	0.070	0.246		
**DMARDs**	0.133	0.186		
**Biologics**	-0.053	0.604		
**Steroids**	0.130	0.198		
**NSAIDs**	0.205	041 [Sig.]		

BMI: body mass index, BASDAI: Bath Ankylosing Spondylitis Disease Activity Index, BASFI: Bath Ankylosing Spondylitis Functional Index, ESR: erythrocytes sedimentation rates, CRP: C-reactive protein, HB: hemoglobin, WBC: white blood cells, N/L ratio: neutrophil/lymphocytes ratio, PLT: platelets, PLT/L: platelets/lymphocytes, DMARDs: disease modifying antirheumatic drugs, and NSAIDs: nonsteroidal anti-inflammatory drugs.

## Data Availability

The data used to support the findings of this study are available from the corresponding author upon request.
